# Role and Evolution of the Extracellular Matrix in the Acquisition of Complex Multicellularity in Eukaryotes: A Macroalgal Perspective

**DOI:** 10.3390/genes12071059

**Published:** 2021-07-10

**Authors:** Bernard Kloareg, Yacine Badis, J. Mark Cock, Gurvan Michel

**Affiliations:** Sorbonne Université, CNRS, Integrative Biology of Marine Models (LBI2M), Station Biologique de Roscoff (SBR), 29680 Roscoff, France; bernard.kloareg@sb-roscoff.fr (B.K.); yacine.badis@sb-roscoff.fr (Y.B.); cock@sb-roscoff.fr (J.M.C.)

**Keywords:** extracellular matrix, cell wall, hydric and salinity stress, biomechanical properties, development, innate immunity, marine macroalgae, evolutionary convergences, multicellularity, sulfated polysaccharides

## Abstract

Multicellular eukaryotes are characterized by an expanded extracellular matrix (ECM) with a diversified composition. The ECM is involved in determining tissue texture, screening cells from the outside medium, development, and innate immunity, all of which are essential features in the biology of multicellular eukaryotes. This review addresses the origin and evolution of the ECM, with a focus on multicellular marine algae. We show that in these lineages the expansion of extracellular matrix played a major role in the acquisition of complex multicellularity through its capacity to connect, position, shield, and defend the cells. Multiple innovations were necessary during these evolutionary processes, leading to striking convergences in the structures and functions of the ECMs of algae, animals, and plants.

## 1. Introduction

**Figure 1:**
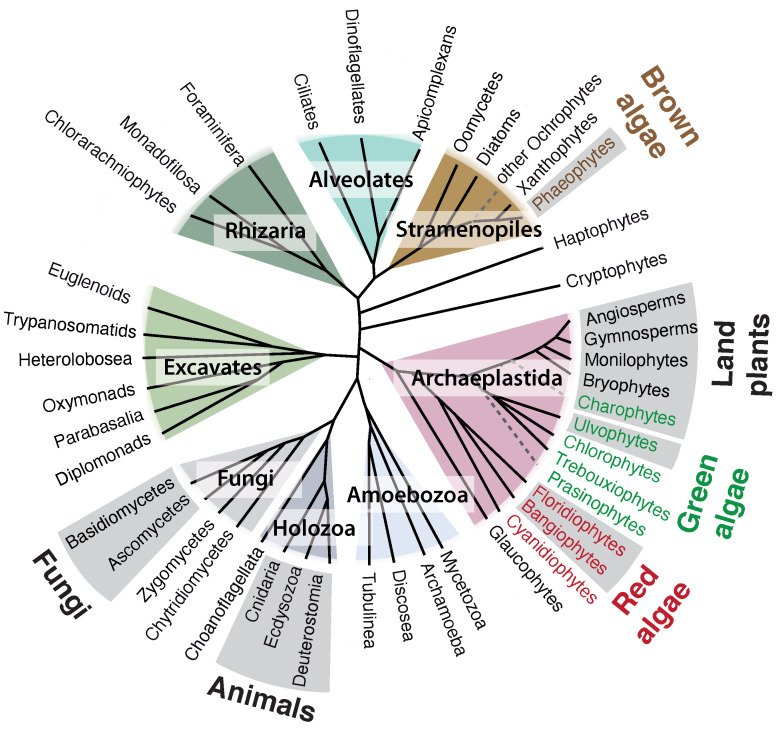
**Schematic tree of the eukaryotes showing the phylogenetic positions of the brown, green, and red macroalgal lineages.** Gray sectors mark lineages that have given rise to complex multicellular species. Adapted from Coelho and Cock, 2020 [3].

Multicellularity has evolved at least 25 times during the evolutionary history of the eukaryotes [1] but only a handful of these multicellular lineages include what could be considered to be complex multicellular organisms. Here, we define complex multicellularity as the possession of a macroscopic body plan consisting of multiple cell types that is constructed by developmental programs involving cell division and differentiation [2]. Under this definition, animals, land plants, and fungi are considered to exhibit complex multicellularity but also the three independently evolved lineages of macroalgae: brown, red and green algae (Figure 1).

A crucial step in the acquisition of multicellularity was the development of an adherent extracellular matrix (ECM), allowing for the transition from cellular autonomy to cellular cooperation. ECMs are complex supramolecular networks that provide multicellular tissues with both rigidity and flexibility. In addition to this structural role, the ECM has important functions in the regulation of development as well as in shielding cells from the outside medium, including protection against abiotic and biotic stresses.

ECMs have been studied intensely in several eukaryotic lineages including animals, fungi, and terrestrial plants, but are less well characterized in the three macroalgal lineages. Here, we review the main features of the organization and functions of the ECM in multicellular eukaryotes, with a particular focus on model macroalgae as these organisms provide a different perspective on the evolution of the ECM and its relationship to the acquisition of complex multicellularity. We revisit our phylogenomic analysis of the evolution of ECM polysaccharides in eukaryotes published ~10 years ago [4]. Over the past decade, considerable progress has been made in our understanding of the functions of the ECM in development and the innate immunity of marine macroalgae. In particular, complete genomes are now available from every macroalgal phylum, notably the brown alga *Ectocarpus siliculosus* [5], the carrageenophyte red alga *Chondrus crispus* [6], the agarophyte red alga *Porphyra umbilicalis* [7], the green alga *Ulva mutabilis* [8], and the charophyte green alga *Chara braunii* [9]. Many of the advances we will discuss have been based on analysis of these reference genomes. There are striking similarities between the functions of the ECMs of algae, animals, plants, and fungi. However, it appears that these are essentially convergent features, which emerged independently in each of the phyla that evolved complex multicellularity.

## 2. Structure of the Extracellular Matrix of Marine Macroalgae

The structure of the ECM has been extensively studied in animals, fungi and plants (see, for review, in [10,11,12,13]). Briefly, animal ECMs are mainly composed of structural proteins (collagens, elastin and fibrillin, laminins, etc.), proteoglycans (core proteins carrying large glycosaminoglycan (GAG) chains such as heparan sulfate (HS), heparin (Hep), chondroitin sulfate (CS), keratin sulfate (KS) and dermatan sulfate (DS), and free glycosaminoglycans (hyaluronan, HA) [10]. Plant ECMs are less flexible and are commonly referred to as the cell wall (CW). These CWs are dominated by semicrystalline cellulose fibers which act as the principal scaffolds for the other CW components. Hemicelluloses (e.g., xyloglucans, glucuronoarabinoxylans, mixed-linked glucans, and mannans) are cross-linking glycans which form hydrogen bonds with cellulose microfibrils. The other main components are the highly complex pectins which constitute a porous matrix embedding the other components. CW proteins, such as extensins and arabinogalactan proteins (AGP), are less abundant than CW polysaccharides but play crucial structural and biological roles [11,12]. In fungi, most cell walls are bi-layered, with the innermost layer made of a core of branched β-1,3-1,6 glucans and chitin, while the outer layer is more variable in composition, with such components as mannan, galactomannan, or β-glucans [13]. In comparison to animals, fungi, and plants, the ECMs of marine macroalgae have been less well characterized although significant progress has been made in recent decades. Like plants, all macroalgae produce cellulose, but these crystalline fibers account for only a small proportion of the cell wall (1–8% of the dry weight) [14,15]. Algal ECMs are dominated by matrix polysaccharides, which greatly vary in nature according to phyla and even lower taxonomic ranks. Notably all marine macrophytes (macroalgae and marine angiosperms) have sulfated polysaccharides in their ECMs [16]; this is in stark contrast with land plants, which do not possess such sulfated glycans. 

**Figure 2:**
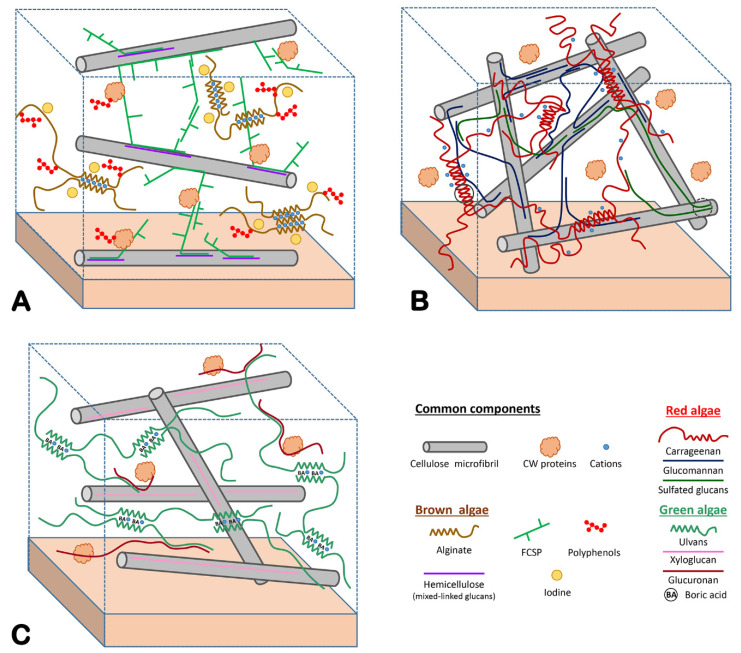
**Models of macroalgal extracellular matrices.** Semicrystalline cellulose microfibrils are present in almost all types of macroalgae. They constitute a crucial structural component but are sparse in comparison to plant cell walls. Most cell wall structural proteins have yet to be characterized, but their identity is expected to differ in each macroalgal phylum. (**A**) Brown algae (example of a Fucales species). Alginates are the most abundant component of brown algal ECMs. Alginate chains auto-associate through their guluronate (G)-rich regions. This process is coordinated by Ca^2+^ ions. Fucose-containing sulfated polysaccharides (FCSPs) acts as cross-linkers between cellulose microfibrils. Hemicelluloses (β-1,4-1,3-glucans, β-1,3-glucans) have been recently identified in brown algae and might act as intermediates between the cellulose microfibrils. Phlorotannins (polyphenols) are likely to be associated with alginates and proteins. Iodine is generally abundant in brown algal ECM but its association mode with other components is unresolved. Arabinogalactan proteins and proteins containing WSC-domains have been identified among CW proteins. Model adapted from Deniaud and coworkers [26] and updated based on the following references [27,28,29]. (**B**) Red algae (example of a carrageenophyte). Sulfated galactans (carrageenans in this case) are the most abundant polymers of the red algal ECM. Chains containing 3,6-anhydro-D-galactose units associate through hydrogen-bonding and hydrophobic interactions. The resulting helices further cluster into microfibrils by ionic interactions between their sulfate groups and cations. Glucomannans have been shown to cross-link cellulose microfibrils. Sulfated glucans have been identified as a minor component, but their interactions with the other polysaccharides remain uncertain. Model adapted from the following references [32,33,34]. (**C**) Green algae (*Ulva* species). The major polysaccharide ulvan can form a weak gel in the presence of both boric acid and divalent cations (e.g., Ca^2+^). However, the exact mechanism of chain association is currently not understood. Two minor polysaccharides were also identified. Xyloglucans interact with cellulose, but their role as cross-linkers of cellulose microfibrils remains to be demonstrated. In contrast, glucuronans interact with ulvans and proteins. Model adapted from Lahaye and Robic [35].

The main CW components of brown algae (Figure 2A) are anionic polysaccharides, namely, alginates and fucose-containing sulfated polysaccharides (FCSPs). Alginates consist of β-1,4-D-mannuronate (M) and its C5-epimer α-1,4-L-guluronate (G), arranged in three types of blocks (MM, GG and MG) along the polysaccharide chain. These uronic polysaccharides form gels in the presence of divalent cations and their rheological properties depend on the relative proportion of GG blocks [17]. FCSPs include two families of highly complex and diverse polysaccharides: sulfated fucans and fucoidans. Sulfated fucans are homopolymers of L-fucose displaying numerous substitutions (e.g., ester-sulfates, acetyl, branching), while fucoidans are heteropolymers containing sulfated L-fucose, but whose main chains are constituted by various neutral and/or uronic monosaccharides (e.g., xylofucoglucuromannans, fucoglucuronans, and fucogalactans) [18]. Sulfated fucans from most Fucales display a backbone with long stretches of alternating α-1,3- and α-1,4-linked L-fucose residues harboring one and two sulfate groups, respectively [19,20,21]. In contrast, the sulfated fucans from Laminariales and Ectocarpales are simpler polysaccharides with only α-1,3-linked L-fucose residues harboring one sulfate group in C4 [22,23,24,25]. Interestingly, *Himanthalia elongata* is unique among Fucales as its sulfated fucan contains highly regular repeating structures with only sulfated α-1,3-linked L-fucose residues, reminiscent of the Laminariales and Ectocarpales sulfated fucans [26]. Recently, hemicelluloses have been identified in brown algal cell walls: β-1,3 glucans [27] and semicrystalline β-1,3-1,4-glucans [28]. Another important finding is the discovery of arabinogalactan-proteins (AGP) in the cell walls of *Fucus* embryos. AGP epitopes were subsequently shown to be present in all the taxonomic groups of brown algae investigated, including the model alga *E. siliculosus* [29]. Finally, brown algal ECMs also contain non-carbohydrate and non-proteinaceous components: phlorotannins (polyphenols) [30] and a high concentration of iodine [31].

The large majority of red macroalgae produce sulfated galactans, agars or carrageenans, which are the main components of their ECM (Figure 2B) [36]. These polysaccharides consist of a backbone of galactose residues linked by alternating β-1,4 and α-1,3 glycosidic bonds. A unique feature of these galactans is the presence of α-3,6-anhydro-galactose moieties (A) in the chain, this remarkable bicyclic monosaccharide being pivotal for the gelling properties of agars and carrageenans. The α-linked galactose units are in the L configuration in agars (L) and in the D configuration in carrageenans (D), whereas the β-linked galactose units are in the D-configuration (G) in both type of polymers [37]. The regular structure of red algal galactans is generally modified by various chemical groups, such as ester sulfate (S) or pyruvic acid acetal groups [38,39]. A frequent modification in agars is the sulfation on the O6 of the L-galactose residues resulting in α-L-galactose-6-sulfate (L6S). For instance, in the agar extracted from *Porphyra* species (commonly named porphyran), the porphyranobiose repeating unit (G-L6S) is twice more abundant than the agarobiose unit (G-LA) [40]. Carrageenans are usually even more sulfated and are classified according to the number and the position of sulfate groups. The main disaccharide repeating units of the three most industrially exploited carrageenans are kappa-carrabiose (G4S-DA), iota-carrabiose (G4S-DA2S), and lambda-carrabiose (G2S-D2S6S) [41]. Whereas commercially processed polymers are mainly composed of one type of repeating unit, native agars and carrageenans have complex hybrid structures. In the last decade, structural characterization of these complex galactans using specific enzymes from marine bacteria (e.g., carrageenases, agarases, and porphyranases) [42,43,44] in combination with NMR and mass spectrometry methods have unraveled novel modifications, such as methylation, presence of uronic acids, or even the presence of neutral monosaccharides as side chains [45,46,47,48,49,50]. Less abundant CW components have been also identified in red macroalgae. Cellulose fibrils are the most commonly found polymer, but additional polysaccharides such as semicrystalline β-1,4-D-mannans, β-1,4 or β-1-3-D-xylans, glucomannans, sulfated xylomannans, and sulfated-mixed-linked glucans have been reported in various red algal species or life cycle stage [15,33,51].

Species from the *Ulva* genus are the most studied green macroalgae. Commonly known as sea lettuce, *Ulva* species are edible algae which are common in coastal ecosystems and typically have a high growth rate and productivity. Unfortunately, due to excess of nutrients released by human activities, these green macroalgae can proliferate, resulting in huge biomass accumulations on seashores (green tides). The *Ulva* ECM (Figure 2C) includes four types of polysaccharides: semicrystalline cellulose, water-soluble ulvans, and two minor hemicelluloses, a xyloglucan and a glucuronan [52]. The interactions between these polysaccharides have been summarized in a model proposed by Lahaye and coworkers [53]. Ulvan is the main CW polymer, representing up to 30% of the algal dry mass [35]. The backbone of this complex sulfated polysaccharide is mostly comprised of L-rhamnose-3-sulfate (Rha3S), D-glucuronic acid (GlcA), its C5-epimer L-iduronic acid (IduA), and D-xylose (Xyl) [54,55,56]. The main chain is mostly made up of disaccharide repeating units comprised of α-L-rhamnose-3-sulfate 1,4-linked to a β-D-glucuronate, a α-L-iduronate or a β-D-xylose. The main repeating units are referred to as type A ulvanobiuronic acid (β-D-GlcA-(1,4)-α-L-Rha3S, symbol: A_3S_), type B ulvanobiuronic acid (α-L-IdoA-(1,4)-α-L-Rha-3S, symbol: B_3S_) and ulvanobiose 3-sulfate (β-D-Xyl-(1,4)-α-L-Rha-3S, symbol: U_3S_) [35]. The determination of the detailed structure of ulvans remain a challenge but specific degrading enzymes (ulvan lyases) have been identified [57,58,59,60,61] and used in combination with NMR and mass spectrometry to characterize ulvan oligosaccharides. A recent major breakthrough was the extensive characterization of the ulvan catabolic pathway in a marine bacterium [62], which provided novel enzyme families (e.g., sulfatases, debranching enzymes) for the structural analysis of this complex polysaccharide. Glycoproteins are also found in *Ulva* ECM [35], but they are essentially uncharacterized.

The biosynthetic pathways for ECM components in marine macroalgae remain largely hypothetical. The function of only a few enzymes have been experimentally validated. In brown algae, a GDP-mannose dehydrogenase converts GDP-mannose into GDP-mannuronic acid (alginate precursor) [63] and mannuronan C5-epimerases catalyzes the C5-epimerization of D-mannuronic acid into L-guluronic acid at the polymer level [64,65]. A type III polyketide synthase is responsible for the synthesis of phoroglucinol, the precursor of brown algal phlorotannin [66]. In red algae, the only biosynthetic step characterized is the conversion of galactose-6-sulfate into 3,6-anhydro-galactose by galactose-6-sulfurylases (in *Porphyra* sp. [67,68] and in *C. crispus* [69] for the biosynthesis of agars and carrageenans, respectively). Nonetheless, this field has seen crucial advances with the sequencing of the complete genome of model macroalgae [5,6,7,8]. Based on these genomic data, potential biosynthetic pathways have been proposed for CW polysaccharides of brown [4] and red algae [6,7,16]. Potential CW-related proteins of *Ulva* have been listed in a supplementary table of the sea lettuce genome article [8], but the biosynthesis of ECM components was not discussed by the authors and a thorough analysis remains to be done for green macroalgae.

## 3. Origin of ECM Components in Plants and Algae

When did the constituents of ECMs arise during evolution? For the polysaccharide constituents, a way to address this question is to look at the phylogeny of enzymes involved in the last steps of their biosynthesis [4]. Below we discuss the case of three major polysaccharides of the ECMs of plants and algae: cellulose/hemicelluloses, sulfated glycans, and alginates.

Cellulose synthases (CESA) and cellulose synthase-like (CSL) proteins are integral membrane enzymes belonging to the family 2 of glycosyltransferases (GT2) [70,71]. While CESA exclusively synthesizes cellulose, CSL-proteins produce different hemicelluloses: mannan (CSLA) [72], xyloglucan (CSLC) [73], β-1,4-glucans (CSLD) [74], and β-1,3-1,4-glucans (CSLF and CSLH) [75,76]. Prior to the sequencing of complete macroalgal genomes, cellulose synthases had been already characterized in the red algae *Pyropia yezoensis* [77] and *Griffithsia monilis* [78]. Homologs of CESA were subsequently identified in the genomes of *E. siliculosus*, *C. crispus*, and *P. umbilicalis*, and their phylogenetic relationships with CESA and CSL of other organisms have been analyzed [4,6,7]. Using as a query the characterized cellulose synthase from *Arabidopsis thaliana* (CESA1, O48946), we have identified here two distant homologues in the genome of the green macroalga *U. mutabilis*: UM008_0140.1 (27% identity on 237 residues) and UM068_0016.1 (25% identity on 240 residues).

**Figure 3:**
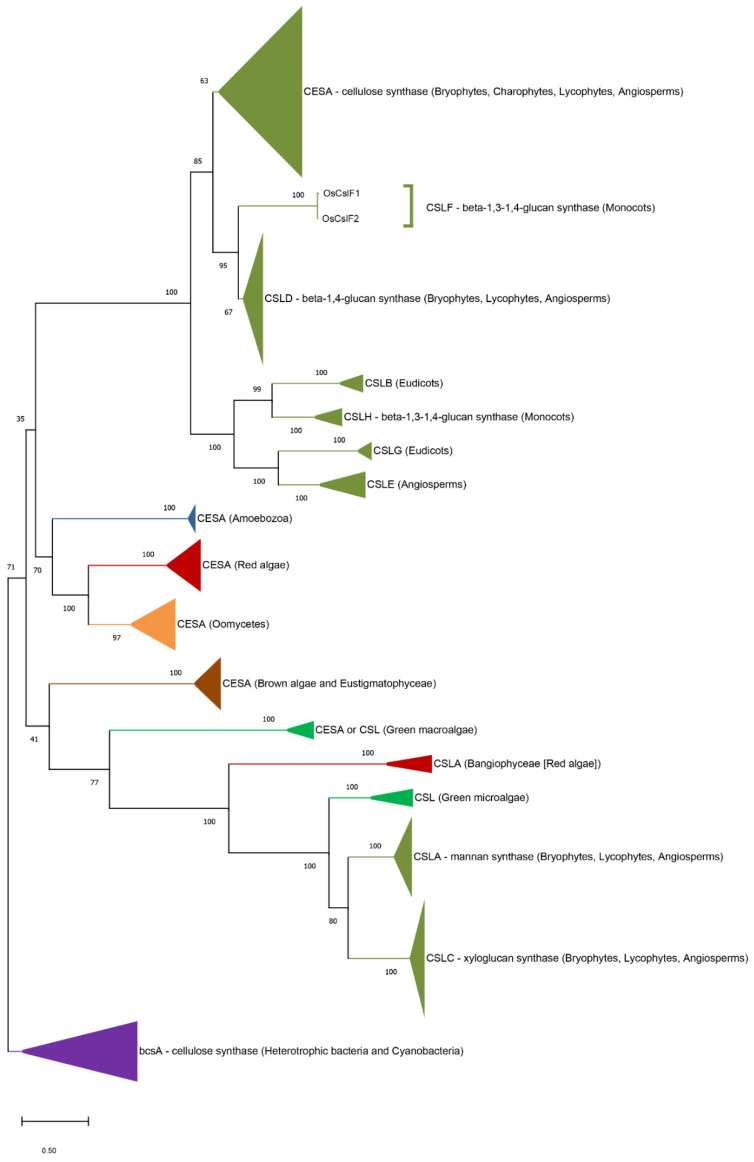
**Phylogenetic tree of the cellulose synthases (CESA) and cellulose synthase-like proteins CSL (family GT2).** The phylogenetic tree was constructed using the Maximum Likelihood (ML) approach with the program MEGA-X [82]. Numbers indicate the bootstrap values in the ML analysis. Bootstrap values inferior to 50% are not shown. The tree was rooted by the clade of bacterial cellulose synthases. The full listing of the aligned proteins is reported in Supplementary Table S1. The uncompressed tree is shown in Supplementary Figure S1.

The origin and evolution of cellulose synthases are particularly relevant for understanding ECM evolution. In numerous bacterial phyla, cellulose is synthesized as an exopolysaccharide. In contrast, the distribution of cellulose (and cellulose synthases, CESA) is very patchy in eukaryotes. Nobles and coworkers were the first to propose a bacterial origin of CESA in land plants, more specifically a cyanobacterial origin suggesting an acquisition during the primary endosymbiosis [79,80]. Nonetheless, with the availability of additional bacterial CESA sequences, it has become clear that cyanobacterial CESA cluster with other bacterial CESA, and thus the exact nature of the ancestral bacteria involved in these horizontal gene transfers (HGT) is very difficult to resolve [4]. Previous phylogenetic analyses of CESA and cellulose synthases like proteins (CSLs) indicated that CESAs from plants, red algae and brown algae have distinct origins, most likely involving independent HGTs with bacteria [4,6,7]. An updated phylogenetic tree including the CSL from *U. mutabilis* is shown in Figure 3. This tree is consistent with previous analyses and is described in more details in the following paragraph. The CESA and CSL proteins from plants diverged into two unrelated clades. The first clade encompasses CESA and CLSB, D, E, F, G and H. The genuine cellulose synthases (CESA) derive from the single cellulose synthase of ancestral Charophyta (freshwater green algae). These genes were duplicated in land plants and the resulting genes evolved to acquire novel activities, giving rise to the different groups of CSL proteins [81]. The second clade, which includes CSLA and CSLC, originated from a CSL gene that has been retained in extant chlorophyte green microalgae but not in Charophytes [9].

Red algal and oomycete CESAs form a cluster distinct from land plant CESAs. Therefore, the CESAs from red algae and plants have different origins, likely independent horizontal gene acquisitions from bacteria. The red algal and oomycete CESAs cluster with the CESA from Amoebozoa, but the node is weakly supported and the position of the Amoebozoa CESA remains uncertain [6]. The clustering of red algal and oomycete CESAs is consistent with the theory of the secondary plastid endosymbiosis, with an acquisition of cellulose biosynthesis by the ancestor of oomycetes through a red algal endosymbiont [6]. Surprisingly, the CESA from brown algae and Eustigmatophyceae (a group of unicellular algae closely related to brown algae) do not cluster with the cellulose synthases from oomycetes, although these organisms all belong to the Stramenopile phylum. This topology was already seen in the phylogenomic study of carbohydrate metabolism of the brown alga *E. siliculosus*, but at that time the lack of genomic data on red macroalgae left open the possibility that they possessed a different type of red algal cellulose synthase closer to the CESA from brown algae [4]. The investigation of *Chondrus* and *Porphyra* genomes clearly invalidates this hypothesis. Therefore, the common ancestor of brown algae and Eustigmatophyceae probably lost the cellulose synthase inherited from the rhodobiont and independently acquired a CESA from an undetermined bacterium. Analysis of the *Porphyra* genome has also provided a major insight into the evolution of hemicellulose biosynthesis [7]. Red algal CSLA (mannan synthase) and CSLC (xyloglucan synthase) were previously thought to be inherited from a common ancestor with the green lineage [81,83], as green microalgae have a closely related CSL, with *Ostreococcus* as the earliest diverging lineage. However, the cell wall of the gametophytes of Bangiophyceae (red macroalgae) is composed of β-1,4-mannans, and two CSL-proteins from *P. umbilicalis* emerge at the root of the plant CSLA/Cs and the green algal CSLs (Figure 3). These enzymes are excellent candidates for CSLA mannan synthases, shifting the origin of CSLA, and thus of mannan, back to the last common ancestor of green and red algae [7]. Thus, mannans may have played a crucial function in the ECM of the Archaeplastida common ancestor. Interestingly, no CSLA gene was found in *C. crispus* genome, consistent with the absence of mannan in this member of Florideophyceae [15]. This suggests that some Florideophyceaean red algae lost the mannan biosynthetic pathway, possibly after the acquisition of the biosynthesis of cellulose, a polymer more crystalline and resistant than mannan. Finally, our phylogenetic analysis indicates that the *Ulva* CESA homologues do not cluster with genuine plant cellulose synthases (CESA). The green macroalgal proteins emerge at the root of the CSLA/CSLC clade, but closer to the CSL from Bangiophyceae than the CSL from plants and green microalgae (Figure 3). Considering the moderate bootstrap value (77%), it is unclear whether the *Ulva* enzymes are involved in the biosynthesis of cellulose or of hemicelluloses (e.g., xyloglucan). In any case this phylogenetic analysis confirms that the origin of cellulose is polyphyletic in the Archaeplastida (red algae, green algae, and plants).

**Figure 4:**
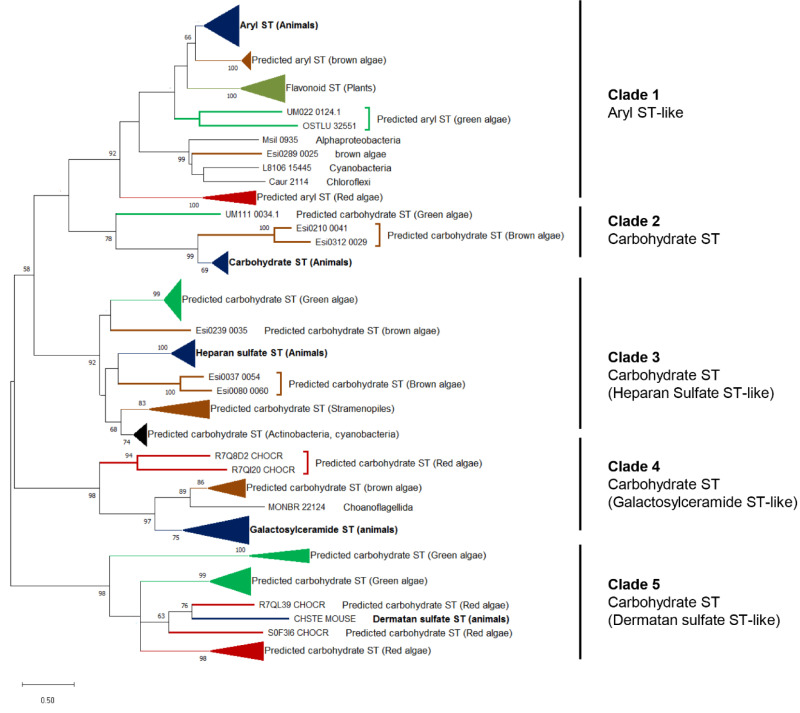
**Unrooted phylogenetic tree of eukaryotic sulfotransferases.** The phylogenetic tree was constructed using the Maximum Likelihood (ML) approach with the program MEGA-X [82]. Numbers indicate the bootstrap values in the ML analysis. Bootstrap values inferior to 50% are not shown. The sequence labels in bold correspond to biochemically characterized sulfotransferases. The full listing of the aligned proteins is reported in Supplementary Table S1. The uncompressed tree is shown in Supplementary Figure S2.

Carbohydrate sulfotransferases (CSTs) are key enzymes for the biosynthesis of sulfated polysaccharides and have been extensively studied in animals [84,85]. Analysis of the *E. siliculosus* genome revealed the presence of both arylsulfotransferases (ASTs) and carbohydrate sulfotransferases in brown algae. These brown algal CSTs are most likely specific for FCSP and are phylogenetically related to animal CSTs involved in the biosynthesis of GAGs [4], suggesting that the capacity to sulfate polysaccharides is an ancestral eukaryotic trait. In contrast, freshwater and land plants only possess AST, which is responsible for the sulfation of secondary metabolites. It has been proposed that the ancestor of land plants lost CSTs, and thus sulfated polysaccharides, during the transition from marine to freshwater environments as a way to adapt to the limited amount of sulfate in terrestrial environments [4]. The ancestral eukaryotic character of CSTs was further highlighted by the identification of sulfotransferases homologous to animal CSTs in the red macroalgae *C. crispus* [6] and *P. umbilicalis* [7], confirming the existence of CSTs in the Archaeplastida phylum. The recent sequencing of the genomes of the marine chlorophyte green alga *U. mutabilis* and of the freshwater charophyte green alga *C. braunii* provide essential missing data and therefore a more complete view on the evolution of CSTs in Archaeplastida. While sulfotransferases were not mentioned in the *Chara* study [9], such enzymes were indicated in a supplementary table of the *U. mutabilis* article [8], but without distinguishing ASTs and CSTs. We have thus searched for CSTs in these green algal genomes. As previously predicted [4], we did not find any CST homologues in the *Chara* genome. To clarify the substrate specificity of the *Ulva* sulfotransferases, we have undertaken a phylogenetic analysis of eukaryotic sulfotransferases (Figure 4).

*U. mutabilis* and the green microalga *Ostreococcus* both possess one putative arylsulfotransferase (UM022_0124 and OSTLU_32551, respectively) related to plant flavonoid sulfotransferases and animal arylsulfotransferases. All the other *Ulva* sulfotransferases are located within three different clades which include characterized animal CSTs (chondroitin ST, heparan ST and dermatan ST, respectively). Therefore, with the exception of UM022_0124, all *Ulva* sulfotransferases are CSTs, most likely involved in the biosynthesis of ulvan. Altogether, these analyses solidly support the hypothesis that ECM sulfated polysaccharides were present in the last common eukaryotic ancestor (with CSTs present in extant animals and marine green, red and brown algae) and that CSTs were lost in the green lineage (charophyte and land plants) as an adaptation to sulfate-scarce freshwater and terrestrial environments. Fascinatingly, seagrasses evolved from an ancestral terrestrial flowering plant which moved back to the sea, and they have sulfated galactans in their cell walls, highlighting again that the presence of sulfated polysaccharides in the ECM is a key adaptation to the marine environment. The genome of *Zostera marina* [86] does not contain a classical CST, confirming that ancestral plants have indeed definitely lost CSTs during the conquest of terrestrial environment. The most parsimonious hypothesis explaining the reacquisition of sulfated polysaccharides by seagrass cell walls is that one or several aryl sulfotransferases, identified in the *Z. marina* genome, evolved in substrate specificity and became able to sulfate carbohydrate [86].

Phylogenomic analyses of *E. siliculosus* support the hypothesis that alginate biosynthesis has a hybrid origin. The first steps, which convert D-fructose-6-phosphate into GDP-mannose, have an ancient eukaryotic origin. In contrast, the alginate-specific steps (catalyzed by GDP-mannose-6-dehydrogenase, mannuronan synthase, and mannuronan C5-epimerases) were acquired by horizontal gene transfer (HGT) from an ancestral actinobacterium [4]. Evidence for a HGT between an actinobacterium and the common ancestor of brown algae is not limited to alginate metabolism. Indeed, *E. siliculosus* possesses a second clade of GT2 family CSL-proteins robustly rooted with the actinobacterial GT2 clade, suggesting the presence of hemicellulose-like polysaccharides of actinobacterial origin [4]. Similarly, phylogenetic analyses indicate that the brown algal capacity to synthesize mannitol and phloroglucinol (phlorotannin precursor) was also acquired from Actinobacteria [66,87]. None of these pathways are found in diatoms nor oomycetes indicating that these HGTs occurred after the divergence of brown algae from the other stramenopiles. This evolutionary event with Actinobacteria had a major impact on the acquisition of a complex multicellularity in brown algae as it provided the main gel-forming polysaccharide in this lineage (alginate), some new hemicellulose-like material, the precursor of a non-carbohydrate ECM component (phlorotannins) and a crucial storage compound, mannitol, which is the carbon translocation form in extant brown algae [4,87]. These HGTs were therefore essential for the emergence of the morphological diversity of extant brown algae and the ecological success of this stramenopile lineage [26,88].

A general conclusion from this overview of CW structure is that it appears that both the skeletal and mucilage components of the ECM of multicellular eukaryotes are not only made of very different materials, essentially proteins and proteoglycans in metazoans and polysaccharides in the photosynthetic eukaryotes, but also of completely different evolutionary origins. Only the capacity to add [4] sulfate substituents to the carbon backbones of carbohydrates was conserved throughout evolution, with the exception of terrestrial algae and plants.

## 4. Biomechanical Properties of the ECM

One important function of the ECM is to connect cells and build tissues and organs. This relies on the biomechanical properties of the extracellular 3-D network of skeletal and matrix macromolecules. The self-assembly and functional properties of gel-forming polysaccharides such as pectins, alginates, agars, and carrageenans were extensively studied in the decades from 1960 to 1990 in the context of the food industry. It is well established that the transition from aqueous solutions to gels is based on coil-helix conformational transitions, which lead to the establishment of junction zones with pseudo-crystalline structures. These consist of aggregation of the carbon chains in different levels of ordered conformations, from single helix to supramolecular assembly [34,89].

For alginates, gel strength depends on guluronate content and block distribution [90]. Mannuronan C5-epimerases are enzymes that convert mannuronic acid into its C5 epimer L-guluronic acid at the polymeric level [91]. These enzymes control the mechanical properties of brown algal tissues, such as holdfasts, stipe, and blades in kelps [17]. ManC5-E genes have been cloned and expressed from *Laminaria digitate* and *E. siliculosus* [64,65]. The importance of mannuronan C5-epimerases in the biology of brown algae is indicated by the occurrence of large multigenic families in brown algal genomes, i.e., 28 genes in *E. siliculosus* [4,5] and 105–125 genes in *Saccharina japonica* [92,93]. A number of ManC5-E genes were upregulated in *Laminaria digitata* in the presence of oligo-alginate signals, which suggests ECM remodeling following biotic stress [94,95]. 

In the red algae, the capacity of galactans such as agars and carrageenans to form supramolecular quaternary structures is dependent on the presence of 3,6-anhydro bridges in the α-1-3-linked galactose moieties. Rees first demonstrated the presence of an enzyme activity in *P. umbilicalis* that removes the sulfate substituents from L-galactose-6-sulfate in porphyran and forms anhydro rings between C3 and C6, characteristic of agarose [67,96]. These enzymes, referred to as galactose-6-sulfurylases, were also identified in the carrageenophyte *C. crispus*. Two different galactose-6-sulfurylases, which specifically catalyze the conversion of nu-carrageenan into iota-carrageenan, were purified and cloned [69]. *C. crispus* has 12 paralogous genes that encode galactose-6-sulfurylases, a rare case of a multigenic family in the *C. crispus* genome [6]. This gene family expansion again suggests that galactose-6-sulfurylases are essential enzymes in the biology of red algae. Galactose-6-sulfurylases are predicted to be extracellular and they probably regulate the packaging of sulfated galactans into a dense, three-dimensional network, thus controlling both the rigidity and elasticity of the ECM [69]. Interestingly, 10 galactose-sulfurylase genes were upregulated in *C. crispus* tetrasporophytes compared to male and female gametophytes, a rather counterintuitive result since the tetrasporophytes contain mainly λ-carrageenan, a heavily sulfated galactan devoid of 3,6 anhydro rings [97]. 

In animal ECMs, procollagenases, also referred to as C- and N-procollagen proteinases, are responsible for the removal of the C- and N-propeptides from procollagen, leading to the assembly of collagen fibrils, which are responsible for the tensile strength and the stability of connective tissues [98,99]. In plants, pectins are methylesterified at the C-6 carboxylic groups of galacturonic acid residues. Pectin methylesterases mediate the removal of methyl ester groups from homogalacturonans. This results in the formation of Ca^+^ ionic bridges between the homoglacturonan molecules but also increases their sensitivity to cleavage by polygalacturonases, thereby either strengthening or loosening plant cell walls [100,101]. The mannuronan-C5-epimerases of brown algae and the galactose-6-sulfurylases of red algae fulfill functions similar to animal procollagenases and to plant pectin methylesterases, namely, the modulation of the biomechanical properties of the ECM. Yet, these enzyme families act with completely different mechanisms, epimerization, de-sulfurylation, peptide cleavage and elimination of methyl-ester groups, respectively. Similarly, the substrates of these processes were acquired independently by brown algae [4], red algae [6], animals, and land plants [102], including charophytes [103] and mosses [104].

## 5. Interfacing with the External Medium

In the ECM of animals, glycosaminoglycans such as chondroitin sulfate [105], keratin sulfate [106], and hyaluronan [107] are highly hydrophilic molecules that confer turgor and flexibility to organs such as cartilage and skin. This is an intrinsic property of their polyanionic nature. Negatively charged polysaccharides provide a dense network of carbon chains with fixed sulfate or carboxylic groups, which are electrochemically compensated by the presence of large amounts of mobile cations, contributing to a net osmotic pressure in the tissues [105]. 

Similarly, algal matrix polysaccharides, which consist of negatively charged macromolecules that carry OSO_3_^−^ or COO^−^ groups, or both, are hygroscopic macromolecules, which help prevent desiccation during low-tide conditions [16]. They can represent as much as 50% of thallus mass in dry weight [15]. The swelling ratio of isolated cell walls from intertidal brown algae in equilibrium with sea water varied from 3.2–9.3, equivalent to approximately 5–15% (*w*/*v*) concentrations of highly ordered, negatively charged molecules. Accordingly, cell walls from intertidal brown algae have cation exchange capacities (CEC) of 3–4 meq.g^−1^ ms [108,109] (Supplementary Table S2). This amounts to a molarity of polyanionic charges of ca 3 M in the ECM of brown macroalgae, a feature necessary to cope with the high salt concentration of seawater, 0.6 M. Due to Donan equilibrium, condensation of counterions on polyelectrolytes and differences in the selectivity of fucans and alginates, this dense matrix interposes an electrochemical screen between the cells and the outside medium, which modifies the concentration, activity, and distribution of anions and cations at the plasma membrane [110]. In elegant experiments involving electrolyte reservoirs separated by a 2% (*w*/*v*) alginate gel, Kowacz and Pollack demonstrated that no substantial mixing occurred between the reservoirs, leading to the establishment of sustained Na^+^/K^+^ concentration gradients, supporting the development of an electric potential difference and attenuating osmotic pressure. Thus, the ECM can be thought of as an additional perm-selective membrane, which helps maintain electrochemical homeostasis in the cytosol without any pumping activity [111].

**Figure 5:**
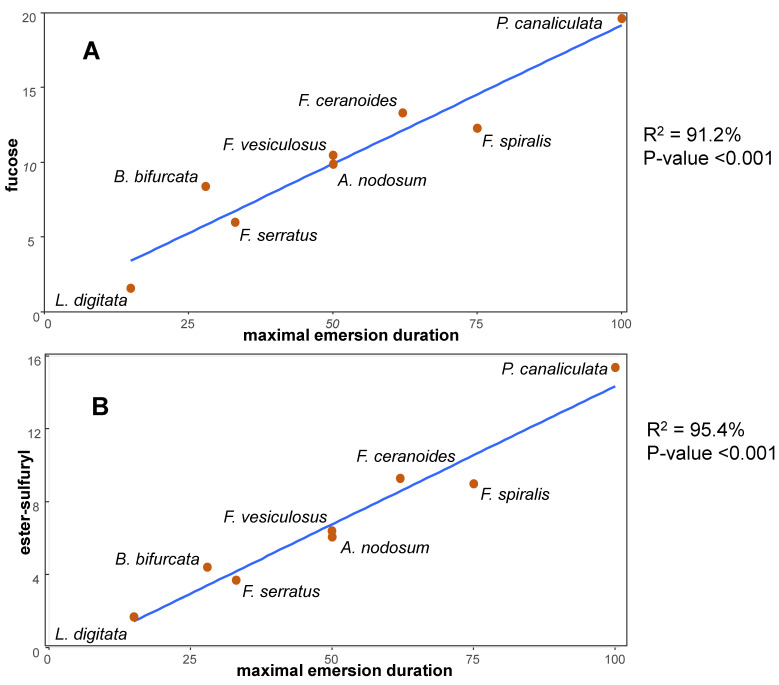
**Correlation between the sulfated fucan content of isolated cell walls of brown algae and their position in the intertidal zone.** (**A**). Fucose content (% *w*/*w*) vs. duration of emersion (% of time). (**B**) Sulfate substituent content (% *w*/*w*) vs. duration of emersion. The maximal duration of emersion in a daily tidal cycle was calculated for Roscoff (Brittany, France), based on species belt zonation in the intertidal (taken as the medium height relative to the lowest astronomical tide), using the spring tide mid-water mark for *Pelvetia canaliculata, Fucus ceranoides, F. spiralis, F. vesiculosus*, and *Ascophyllum nodosum* and the neap tide mid-water mark for *F. serratus*, *Bifurcaria bifurcate*, and *Laminaria digitata*. See Supplementary Table S2 for the corresponding data values.

In intertidal brown algae, a strong correlation was seen between the duration of emersion of individual species and the fucose and sulfate contents of the isolated cell walls (Figure 5). This suggested that the sulfated fucans of the ECM are specifically involved in water and ionic regulation in intertidal brown algae [26,110]. Experimental evidence to support these assumptions was provided by investigating acclimation of *Ectocarpus subulatus* to changes in salinity. *E. subulatus* is an *Ectocarpus* isolate from freshwater that is tolerant to normal seawater, with a morphotype consisting of long filaments. When transferred from freshwater to normal seawater, *E. subulatus* developed star-shaped thali that resembled the morphotype of the marine species, *E. siliculosus*. These morphological changes were paralleled by massive changes in the transcriptome. Notably, two fucan sulfotransferase genes were induced in seawater while fucan sulfohydrolase genes were induced in the freshwater medium [112]. The incorporation of sulfated fucans into the ECM of *E. subulatus* was further investigated using monoclonal antibodies that recognize sulfated fucans, showing that this strain modulates the substitution of sulfate groups on fucans in relation to salinity levels [113]. Siméon and coauthors confirmed that the branching activity of *E. subulatus* filaments depends on the salinity of seawater [114]. However, it is the presence of sulfate in the culture medium, and not the overall ionic strength, that triggers growth of the primary filaments as well at the emergence of secondary filaments. This developmental pattern correlated with the incorporation of sulfated fucans at the dome of apical cells, suggesting that sulfated fucans control the viscoelasticity of the ECM [114]. Other evidence of a correlation between the presence of sulfated polysaccharides and tolerance to salt stress is provided by marine and mangrove angiosperms. For example, the seagrass *Ruppia maritima* synthesizes a sulfated galactan, in amounts of ~1% (*m*/*m*) in both the roots and leaves [115]. When *R. maritima* was cultivated in the absence of salt, it produced no sulfated galactans [116]. The mechanisms by which sulfated polysaccharides help to cope with salt stress remain elusive, however. 

## 6. ECM Signaling and Development

**Figure 6:**
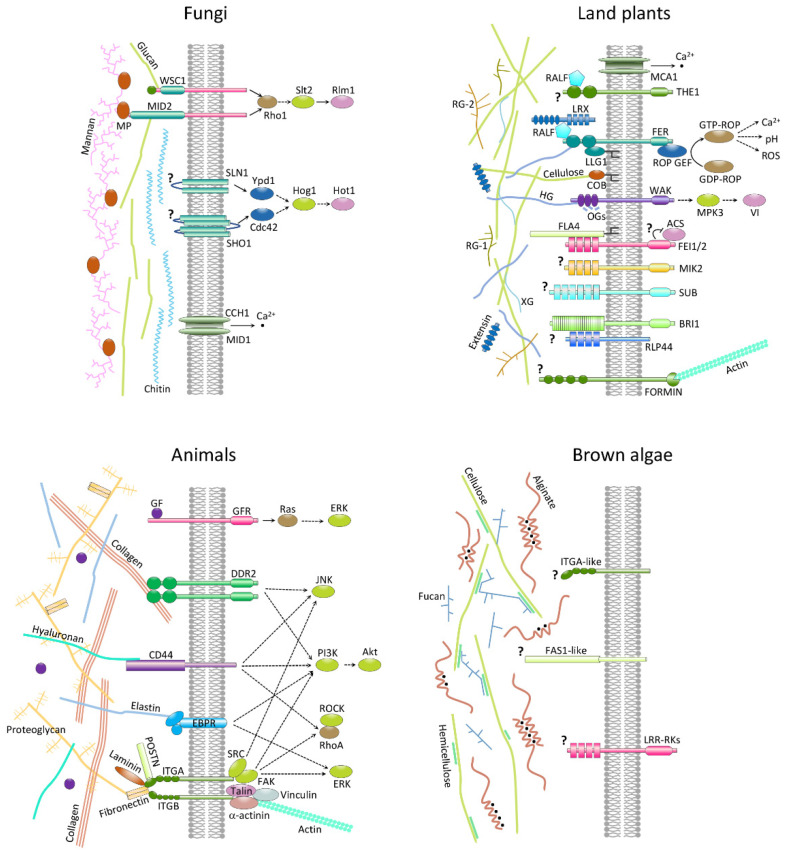
**Membrane-localized, ECM-sensing proteins in land plant, animals, and fungi.** Related membrane proteins identified in brown algae are also shown. MCA1, MIDI1-complementing activity 1; THE1, THESEUS1; FER, FERONIA; WAK, wall-associated kinase; RALF, rapid alkalinization factor; LRX, leucine-rich repeat extensin; LLG1, LORELEI-like glycosylphosphatidylinositol-anchored protein 1; FLA4, fasciclin-like arabinogalactan protein 4; COB, extracellular glycosyl-phosphatidyl inositol-(GPI)-anchored protein COBRA; FEI1/2, leucine-rich repeat receptor kinases; MIK2, MALE DISCOVERER1-interacting receptor like kinase 2 I; SUB, STRUBBELIG; BRI1, brassinosteroid insensitive 1; RLP44, receptor-like protein 44; ROP, rho of plants; ROP GEF, rho of plants guanine-nucleotide exchange factor; MPK3, map kinase 3; VI, vacuolar invertase; ACS, aminocyclopropane 1-carboxylic acid synthase HG, homogalacturonan; OGs, oligogalacturonides; XG, xyloglucan; RG-1, rhamnogalacturonan I; RG-2, rhamnogalacturonan II; ROS, reactive oxygen species; GF, growth factor; GFR, growth factor receptor; DDR2, Discoidin domain-containing receptor 2; EBPR, Elastin-binding protein receptor, ITGA/B, Integrin A/B subunit; POSTN, fasciclin domain protein periostin; Ras, Ras GTPase; JNK, c-Jun N-terminal mitogen-activated protein kinase; PI3K, Phosphoinositide 3-kinase; Akt, RAC-α serine/threonine-protein kinase; RhoA, Ras homolog A GTPase; ROCK, Rho-associated protein kinase; ERK, Extracellular-signal-regulated mitogen-activated protein kinase; WSC1, Cell wall integrity and stress response component 1; MID2, Mating pheromone-induced death protein 2; SLN1, Synthetic lethal of N-end rule 1; SHO1, Synthetic high osmolarity-sensitive 1; CCH1/MID1, Stretch-activated voltage-gated high-affinity calcium channel composed of Calcium channel homolog 1 and Mating pheromone-induced death protein 1; Rho1, Ras homolog 1 GTPase; Slt2, Suppressor of the lytic phenotype mitogen-activated protein kinase 2; Rlm1, Resistance to lethality of MKK1P386 overexpression MADS-box transcription factor 1; Ypd1, Tyrosine phosphatase dependent 1; Cdc42, Cell division cycle 42 rho-like GTPase; Hog1, High-osmolarity glycerol mitogen-activated protein kinase 1; Hot1, High-osmolarity-induced transcription factor 1; MP, mannoprotein; FAS1-like, Fasciclin-like domain protein, LRR-RKs, Leucine-rich repeat domain receptor kinases; ?, ligand unknown or uncertain step of the signal transduction pathway; black dots, calcium atoms; branched black lines indicate GPI anchors. Adapted from [26,117,118].

The ECM has an important influence on growth and development in animals, green plants, and fungi. Cell wall integrity pathways provide a clear illustration of this relationship in that they directly link modifications to the cell wall to cellular responses via signal transduction pathways. Figure 6 illustrates the three different pathways that have been shown to be involved in sensing cell wall integrity in yeast [117]. The transmembrane cell wall stress sensors WSC 1–3 and MTL2/MID2 signal through the small GTPase RHO1 and protein kinase cascades to a number of different downstream transcription factors. SHO1 and the sensor histidine kinase SLN1 are osmosensors that signal via the Hog1 MAPK and, finally, CCH1 and MID1 constitute a stretch activated calcium channel complex that also plays a role in osmosensing. 

In plants, challenges to cell wall integrity, for example, as a result of biotic or abiotic stress, lead to reduced growth rate. Numerous different proteins have been implicated in sensing changes in cell wall integrity in this lineage (Figure 6) [117,118]. These proteins include several families of plant receptor kinase (CrRLK1L, which includes FERONIA and THESEUS1, WAK and LRR), LRR extensins, GPI-anchored proteins (including AGPs), mechanosensing ion channels (MCA1), and proteins that link the cell wall with the cytoskeleton such as FORMIN HOMOLOGY1. In animals, the ECM is the most important source of external signals to cells and is generally considered to be an integral component of many signal transduction pathways [119].

The importance of the ECM to animal cells is illustrated by the phenomenon of anoikis, which is a form of apoptosis that occurs in response to inadequate or inappropriate cell–matrix interactions. Integrins are the main adhesion proteins that bridge the cellular cytoskeleton with the extracellular matrix [120]. Several other proteins interact with ECM components and transmit signals into the cell, including the collagen binding receptor DDR2, the transmembrane proteoglycan receptor CD44 (which binds hyaluronan), elastin binding protein receptors, and growth factor receptors [119,120]. These different ECM-interacting membrane-localized systems are illustrated in Figure 6. 

Signal transduction pathways linking the extracellular matrix to growth and development have not yet been characterized for macroalgae, but there have been several reports that have indicated a role for the extracellular matrix in the regulation of developmental processes. In young (zygote and 2-cell stages) fucoid embryos for example, the extracellular matrix is both essential for the fixation of the polarization axis of the zygote [121] and can direct cells to adopt a rhizoid or thallus cell fate [122]. In older embryos the situation is more complex, and cell fate determination appears to involve both positional information and diffusible intercellular signals [123]. However, even at these later stages, there is evidence that signals between cells transit via the apoplast, indicating an intermediate role for the ECM as the communication medium. In another study, sporophytes of the filamentous brown alga *Ectocarpus* were shown to secrete a diffusible factor into the surrounding medium that could induce gametophyte initial cells to switch to the sporophyte developmental program [124]. The involvement of a secreted factor again implies a role for the ECM. Interestingly, the sporophyte-inducing diffusible factor is only active on initial cells before they synthesize a cell wall [124]. Loss of sensitivity to the factor may simply be the result of the cell wall acting as a physical barrier, but it is also possible that the cell wall is acting in a similar manner to that observed during early development in *Fucus*, in this case transmitting a signal that locks cells into the developmental program they have initiated. At present, none of the molecules involved in these signaling pathways have been characterized biochemically, although recent evidence suggests that the diffusible sporophyte-inducing factor in *Ectocarpus* is a large molecule that may be related to arabinogalactan proteins [125]. 

One approach that has been used to identify potential components of ECM–cell signaling pathways in macroalgae has been to search for homologues of the proteins that carry out these functions in animals, land plants, and fungi (see Figure 6 for some proteins encoded by the *Ectocarpus* genome). For example, brown algae independently evolved a family of membrane-localized receptor kinases with leucine-rich repeat extracellular domains [5], but it is not known if these receptors interact with cell wall components. Red macroalgae, in contrast, do not appear to possess receptor kinases [6,7]. Intriguingly, brown algae also have membrane-localized proteins similar to animal α integrins, together with homologues of some cytosolic integrin interactors (talin and α-actinin but not vinculin) and a fasciclin-like domain protein. However, brown algae lack the ECM components that interact with integrins in animal systems such as collagen, fibronectin, and vitronectin [7], and it remains to be determined whether an integrin-like pathway exists in macroalgae. In addition, genome analysis has identified predicted proteins with arabinogalactan protein backbone motifs, indicating that this class of molecule also occurs in brown algae [29]. Moreover, expression of chimeric AGP-like core proteins has been shown to be developmentally regulated in fucoid embryos and experiments designed to block the action of these proteins inhibited the progression of early embryogenesis [29].

## 7. ECM Signaling and Innate Immunity

As multicellular eukaryotic lineages arose, with longer generation times than their unicellular ancestors, they represented rich nutrient niches for microbes. This led to the natural selection of hosts with efficient innate immunity systems capable of controlling microbial colonization [126]. In both plants and animals, the first line of defense is the recognition of Pathogen/Microbial Associated Molecular Patterns (P/MAMPs) or of Damage/Danger Associated Molecular Patterns (DAMPs) by Pattern Recognition Receptors (PPRs) located at the plasma membrane. PAMPs, formerly referred to as exogenous elicitors, consist of evolutionary conserved molecular motives that distinguish hosts from pathogens. They include bacterial lipopolysaccharides, peptidoglycan, flagellin, as well as fungal glucans and mannans [127]. DAMPs, formerly referred to as endogenous elicitors, consist of degradation products of the host, as evidence of loss of tissue or cellular integrity. In animals, ECM constituents such as hyaluronan, glycosaminoglycans, and fibronectin form bioactive fragments during infection or injury, which activate PRRs such as Toll-Like Receptors (TLRs) both in non-immune cells (e.g., fibroblast and epithelial cells) and in immune cells (e.g., macrophages and dendritic cells), alerting the immune system of tissue damage and infection, which initiates pathogen clearance and tissue repair [128]. In plants, attacks by microbes, fungi, oomycetes, nematodes, and insects also lead to fragmentation of the ECM and the sensing of danger signals such as cellobiose and oligogalacturonides provides a means of detecting challenges to cell wall and tissue integrity [129]. 

In plants, activation of PRRs leads to intracellular signaling, transcriptional reprogramming, and induced responses that limit microbial colonization [130]. These include the expression of so-called Pathogenesis Related Proteins (PRPs) some of which target the ECM of either the host (peroxidases which cross-link cell wall constituents) or the attacker (e.g., lysozyme, glucanases, and chitinases). PAMP-triggered immunity (PTI) controls colonization of non-adapted pathogens, e.g., of commensals and symbionts [126,129,131]. In contrast, host-adapted plant pathogens deploy virulence factors (or effectors) in the host cells which interfere with PTI, increasing host susceptibility. The host, in turn, can perceive such effectors through additional receptors, known previously as R (Resistance) proteins and nowadays as NB-LRR proteins, for Nucleotide-Binding, Leucine-Rich Repeat proteins [132]. When the structure or the action of a given effector is recognized by one of the NB-LRR proteins, the host builds a second layer of defense, referred to as Effector Triggered Immunity (ETI). ETI is an accelerated and amplified PTI response, resulting in disease resistance and, usually, hypersensitive cell death (HR) at the site of infection [130,131,132].

Like terrestrial multicellular eukaryotes, marine macroalgae face a variety of potential attackers, including viruses, bacteria, fungi, oomycetes, grazers, as well as other algae, which can be endophytic or epiphytic [133]. Macroalgae resemble plants and metazoans in their basic mechanisms for non-host recognition, including recognition of P/MAMPs and DAMPs, activation of the oxidative burst machinery, as well as cleavage and peroxidation of polyunsaturated fatty acids [134,135,136]. Sporophytes of the brown algal kelp *Laminaria digitata*, for example, react to the presence of alginate fragments with a rapid burst of reactive oxygen species [137], leading to resistance to invasion by the brown algal endophyte *Laminariocolax tomentosoides* [138]. Oligo-guluronate signals mimic ECM damage following pathogen attack or grazing. They have been used to decipher the signaling and metabolic pathways involved in defense in kelps [95], to demonstrate that kelp sporophytes have systemic defense responses [139] and to show that kelps are capable of distance signaling [140]. 

For the phylum Rhodophyta, direct evidence for the recognition of ECM fragments such as DAMPs has been provided for *Gracilaria conferta* (Gracilariales), based on the accumulation of hydrogen peroxide within minutes in the medium following exposure with agar oligosaccharides [141,142]. Ligand–receptor association appears to be as finely tuned as the recognition of glucan and pectin elicitors by plants: only 0.01–0.5 μM concentrations of the optimally sized agaro-oligosaccharides were necessary to induce half-maximal reactions by *G. conferta* [143]. The association between the red alga *C. crispus* and the filamentous green alga, *Acrochaete operculata* is an interesting system of both host and non-host interactions. As mentioned above *C. crispus* has an isomorphic life history, in which the haploid gametophytic and diploid sporophytic generations differ only by minor traits, such as the sulfate-ester group distribution of their matrix polysaccharides, κ- and λ-carrageenans. Consistent with the ECM composition of *C. crispus* sporophytes (the generation susceptible to invasion by *A. operculata* filaments), λ-carrageenan oligosaccharides stimulate protein synthesis and elicit the production of specific polypeptides in the pathogen, resulting in a marked increase in pathogenicity. In contrast, incubation of the pathogen in the presence of κ-carrageenan oligosaccharides enhances pathogen recognition by the host and reduces the virulence of the pathogen [144]. Induction of PTI in *C. crispus* gametophytes upon incubation with cell-free extracts of *A. operculata* was shown to involve oxylipins synthesized from both C:18 and C:20 polyunsaturated fatty acids [145]. Another example of host recognition through matrix polysaccharides was reported in the Bangiales species *Porphyra yezoensis*. Sulfated agar, known as porphyran, but not agarose, was shown to enhance appressorium formation in the oomycete *Pythium porphyrae*, the agent of red rot disease in this red alga [146].

Development of laboratory-controlled host–pathogen systems and the availability of reference genomes from brown and red macroalgae was essential in further deciphering the molecular bases of innate immunity in marine macroalgae. Among the eukaryotic pathogens of Phaeophyta, the marine oomycete pathogen *Eurychama dicksonii* is the most widespread, with evidence of genetic determinism for disease resistance in brown algae [147]. Using ESTs prepared from cultures of *E. siliculosus* infected by *E. dicksonnii*, Grenville-Briggs and coworkers identified pathogenicity effectors in *E. dicksonnii,* including an alginate lyase, while the host secreted specific cellulase synthases, mannuronan-C5-epimerase and protease inhibitors [148]. Another oomycete, *Anisolpidium ectocarpii*, was used to investigate local defenses and systemic responses in the giant kelp *Macrocystis pyrifera* [149]. In this pathosystem, papillae are formed by the host cell at the site of pathogen penetration, followed by differentiation of a new cell wall underneath the papillae, involving deposition of callose-like β-1,3-glucans [150].

As for the Rhodophyta, a high coverage transcriptome analysis of the Ceramiale *Laurencia dendroica* inoculated with *Vibrio madracius* indicated that LRR proteins potentially involved in non-host recognition, were constitutively expressed in the host while genes coding for defense-related transcription genes, ROS metabolism, and terpene biosynthesis were upregulated [151]. The genus *Pyropia* (Bangiales), the most valuable seaweed aquaculture crop worldwide, has also received a lot of attention. Micro-array profiles of *P. tenera* cultures, infected by its most destructive pathogens, *Phytium porphyrae* and *Olpidiopis pyropiae* (oomycetes) and the virus PyroV1, were investigated together with histochemical observations [152]. Serine protease and cell wall-associated hydrolase genes (likely to be involved in the apoplastic defence) were the most common and abundant pathogen-induced genes. During *O. pyropiae* infections, components reminiscent of effector-triggered immunity (ETI) in plants were highly upregulated, including ubiquitin-containing enzyme [152]. These results were confirmed by analysis of the transcriptome of another *Pyropia* species, *P. yezoensis*, infected by *Pythium porphyrae.* Pattern Recognition Receptors, including three lectin genes and a Toll/interleukin-1 receptor, were upregulated after infection. Expression of genes related to the ubiquitin-proteasome system and hypersensitive cell death, again reminiscent of plant effector-triggered immunity, increased significantly during the infection [153].

**Figure 7:**
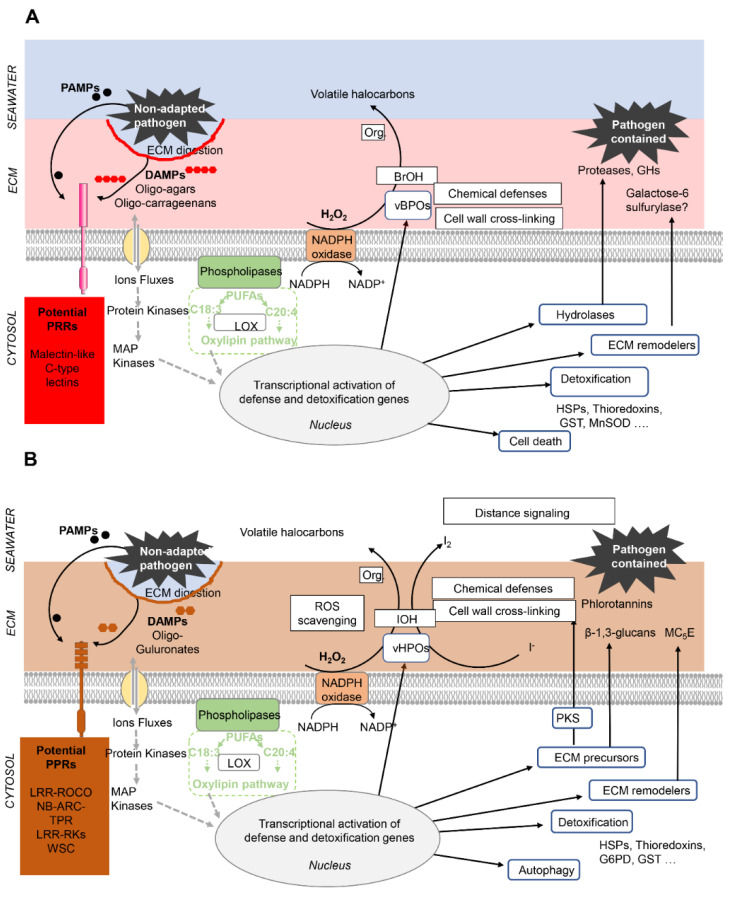
**Role of the extracellular matrix in the innate immunity of multicellular red (A) and brown (B) algae.** Drawings adapted from in [136] and updated based on evidence provided by the authors of [7,139,140,149,152,161]. Note that the basic principles of innate immunity are shared by brown and red algae (as well as by plants and animals): recognition by Pattern Recognition Receptors (PPRs) of pathogen-derived PAMPs or cell-wall derived DAMPs triggers signaling cascades that activate defense and detoxification responses. Red (A) and brown algae (B) differ by the nature of their DAMPs and PPRs, highlighted in red and brown, respectively. In the ECM per se the main defense responses deal with ECM remodeling (thickening, strengthening) and the secretion of chemicals and enzymes that target the pathogen, eventually leading to the containment of non-adapted pathogens. Abbreviations: PAMPs, pathogen associated molecular patterns; DAMPs, Damage associated molecular patterns; PUFAs, poly unsaturated fatty acids; MAP Kinase, mitogen activated protein kinase; LOX, lipoxygenase; vHPO, vanadium-dependent haloperoxidase; IoH, hypoiodous acid; vBPO, vanadium-dependent bromoperoxidase; BrOH, hypobromous acid; ROS, reactive oxygen species. Org. organic compound. HSPs, heat shock proteins; G6PD, glucose-6-phospate-dehydrogenase; GST, Glutathion-S-transferase; SOD, superoxide dismutase; PKS, polyketate synthase; MC5E, mannuronan C5 epimerase, GHs, glycoside hydrolases.

Marine algae have taken advantage of the presence of bromide (3 mM) and iodide (0.3μM) ions in seawater [154] to evolve specific defenses against abiotic and biotic stress [95]. For example, the kelp *L. digitata* concentrates iodine up to 2% of its dry weight [155]. Iodine is stored in the apoplasm, in the form of iodide ions, where it can be easily remobilized for chemical defense and antioxidative properties [31,156]. Halogen metabolism in kelps depends on vanadium-dependent haloperoxidases (vHPOS), including iodoperoxidases that are specific for the oxidation of iodide into highly reactive, hypoiodous acid [157,158]. The *Saccharina japonica* encodes 89 vHPOs, including 21 bromoperoxidases and 68 iodoperoxidases. Such a large gene family size underlines the importance of haloperoxidases in kelp biology [159], e.g., for detoxification and the production of defense compounds [95]. Additionally, oxidative cross-linking was shown to promote the formation of phenolic polymers and their complexation with alginates [160]. This provides a potential functional link between the induction of these general stress marker genes and strengthening of the cell wall (Figure 7). 

The striking similarity between animal NLRs and plant R-proteins raised the question of whether innate immune signaling systems are ancestral to all eukaryotes [127,129]. It is now agreed that, even though individual components of the plant and animal innate immune signaling pathways share some common protein motifs, including TIR, NBS, and LRR domains, these motifs have been recruited independently in animals and plants [127,162,163]. Analysis of brown and red algal genomes support the view that the innate immunity systems also evolved independently in these lineages. The *E. siliculosus* genome contained no clear orthologues of plant resistance genes or animal pathogen receptors. Instead, numerous LRR-GTPases of the ROCO family as well as NB-ARC-TPR proteins were identified [161]. Similarly, analysis of the *P. umbilicalis* genome found no evidence for canonical plant cell surface or intracellular receptors. However, two families of intracellular ligand-binding proteins containing NB-ARC domains and a family of potential extracellular receptors containing malectin and Ig-like fold domains were found in multicellular red algal genomes. A family of potential microbe-sensing domains, specific of the Bangiophytes, was also identified, including C-lectins with Von Willebrand factor type A [7]. Several of these proteins were upregulated in *Pyropia* cultures infected by oomycetes [152,153]. Altogether, innate immunity in marine macroalgae consists of general constitutive defenses and genetic toolkits against specific pathogens [152,161]. The ECM appears as a central battleground for PTI, both in the recognition of PAMPs and DAMPS and in the retaliation against attackers (Figure 7). 

## 8. Conclusions

The physical and chemical constraints of the marine and terrestrial environments are substantially different. For photosynthetic eukaryotes immersion in seawater and being rooted in the soil represent very different conditions in terms of availability of water and nutrients as well as perception of physical and chemical pressures. However, the biological constraints are similar, involving resistance to abiotic and biotic stresses. Transitions from uni- to multi-cellularity have occurred in the context of these physical and biological constraints and have had to take into account these stresses. 

The evidence discussed in the various sections above indicates that—as was also the case for animals, land plants, and fungi—the ECMs of macroalgae not only are important from a structural point of view, providing a matrix that holds cells together within tissues and protecting them from the environment, but they probably also play important roles in signaling events related to multicellular development and defense responses.

When we compare the ECMs of diverse eukaryotic lineages, it is remarkable that only a few elements can be traced back to their unicellular common ancestor. The carbohydrate sulfotransferases (CSTs), which mediate the addition of sulfate-ester groups to matrix sulfated polysaccharides, appear to be very ancient in eukaryotes as they are shared by extant organisms belonging to at least three distant phyla (Opisthokonta (metazoans), Archaeplastida (marine red and green macroalgae), and Stramenopiles (marine diatoms and brown algae). Absence of CSTs from extant charophytes and land plants (Archaeplastida) and oomycetes (Stramenopiles) is most likely due to gene loss as a result of adaptation to freshwater and terrestrial environments, which are poor in sulfate. In contrast, the vast majority of ECM material has been acquired de novo in each multicellular lineage. This is the case for self-associating linear macromolecules that compose the fibrillar components of ECM, such as collagens in metazoans, β-1,3-glucan and chitin in fungi, cellulose and hemicellulose in amoeba, algae and plants. Here, we present evidence that cellulose biosynthesis was acquired independently in all of the photosynthetic lineages and in amoeba by the early recruitment of cellulose synthases from distinct classes of bacteria. The construction of organized parenchymas required the recruitment and the expansion of polymers, such as proteoglycans, pectins, ulvans, sulfated galactans, and alginates, which intersperse and cross-link the semicrystalline fibrillary elements. Again, the synthases, such as glycosyl transferases, which elongate these macromolecular chains are mostly not homologous between these multicellular lineages. Not surprisingly, this is also the case of the enzymes that control the self-assembly of these linear macromolecules and the plasticity of the ECM, i.e., pro-collagenases in animals, pectin methyl-esterases in Viridiplantae, mannuronan-C5-epimerases in brown algae and galactose-6-sulfurylases in red algae.

As the shielding functions of different types of ECM essentially derive from their macromolecular composition and architecture, they can be thought of as clear examples of convergent evolution within the context of the emergence of multicellularity. A similar conclusion can be drawn for the first line of defense in innate immunity, the recognition by extracellular Pattern Recognition Receptors of Pathogen/Microbial Associated Molecular Patterns or of Damage/Danger Associated Molecular Patterns derived from the ECM of pathogens or the host, respectively. The intracellular machinery associated with early responses to biotic and abiotic stress, including NADPH oxidases, phospholipases, and lipoxygenases, is present in all of the multicellular lineages, likely a legacy from the unicellular common ancestor of eukaryotes [145,164,165]. However, and in spite of structural similarities, PPRs were shown to have arisen independently in animals and plants (see, e.g., in [163]) and, although functional evidence is absent, we believe that this is also the case in the macroalgal lineages.

Given the independent evolutionary origins of the ECMs of different eukaryotic lineages, it should also not be surprising that each lineage has independently evolved systems that link ECM integrity and function to developmental processes. This has clearly been the case for animals, land plants and fungi. These processes have yet to be identified and characterized in macroalgae but there are already indications that seaweeds may also conform to this “rule”. For example, although a small number of molecules resembling ECM regulators in other lineages, such as integrin-like molecules and receptor kinases, have been identified in brown algae, it is unlikely that they function in the same manner in this algal lineage. Many of the key components of integrin signaling are absent, suggesting that the integrin-like molecules may be recognizing other ligands and functioning in other pathways. Similarly, receptor kinases appear to have evolved independently in the brown algae [5] and again this would be consistent with the emergence of functional novelty.

The focus on macroalgae here has highlighted the interest and novelty of these model systems but has also underlined the lack of information compared to more intensely studied lineages such as animals, land plants and fungi. This situation is changing with the establishment of more extensive genomic resources (e.g., https://phaeoexplorer.sb-roscoff.fr/home (accessed on the 9 July 2021) and the recent development of genetic tools to explore gene function in macroalgae such as forward genetics [166] and genetic transformation [167,168] and genome editing [167,168]. One particular area of interest to explore with such new approaches is the biosynthesis and trafficking of ECM components. It is most likely that, as in the other lineages of multicellular eukaryotes, ECM assembly in macroalgae requires the coordinated involvement of the nucleus, endoplasmic reticulum, Golgi apparatus, secretion vesicles and the plasma membrane. The molecular tools that are now available should allow this coordinated process to be worked out in detail. With these tools one can also expect to gain further insight into the molecular bases of ECM functions in marine macroalgae. 

## Data Availability

Not applicable.

## Supplementary Materials

The following are available online at https://www.mdpi.com/article/10.3390/genes12071059/s1, The data presented in this study are available in the Supplementary Material (Tables S1 and S2, Figures S1 and S2).
