# Supplementary material for: Role and Evolution of the Extracellular Matrix in the Acquisition of Complex Multicellularity in Eukaryotes: A Macroalgal Perspective
Source: Genes (Basel). 2021 Jul 10;12(7):1059. doi: 10.3390/genes12071059 (PMC8307928; doi:10.3390/genes12071059)
Supplement: Supplementary file 1 [file genes-12-01059-s001.zip › Supplementary files/Supplementary Table S2.pdf]

Supplementary Table S2. Correlation between the sulfated fucan content of isolated cell walls of brown algae and their position in the intertidal zone. Data values corresponding to Figure 4. Height was measured relative to the lowest astronomical tide. CEC, Cation Exchange Capacity. Adapted from [12, 100, 101].

**Species**	**Height (m)**	**Max. emersion (% time)**	**fucose content (% w/w)**	**SO4 content (% w/w)**	**CEC (meq.g^{-1} ms)**
P. canaliculata	7.3	100	19.6	15.4	3.9
F. spiralis	6.1	75	12.3	9.0	3.4
F. ceranoides	5.4	62	13.3	9.3	3.2
F. vesiculosus	4.8	50	10.5	6.4	3.3
A. nodosum	4.8	50	9.9	6.1	3.6
F. serratus	2.3	33	6.0	3.7	3.1
B. bifurcata	1.8	28	8.4	4.4	3.1
L. digitata	0.5	15	1.6	1.7	3.8
