# Supplementary figures and images for: Role and Evolution of the Extracellular Matrix in the Acquisition of Complex Multicellularity in Eukaryotes: A Macroalgal Perspective
Source: Genes (Basel). 2021 Jul 10;12(7):1059. doi: 10.3390/genes12071059 (PMC8307928; doi:10.3390/genes12071059)

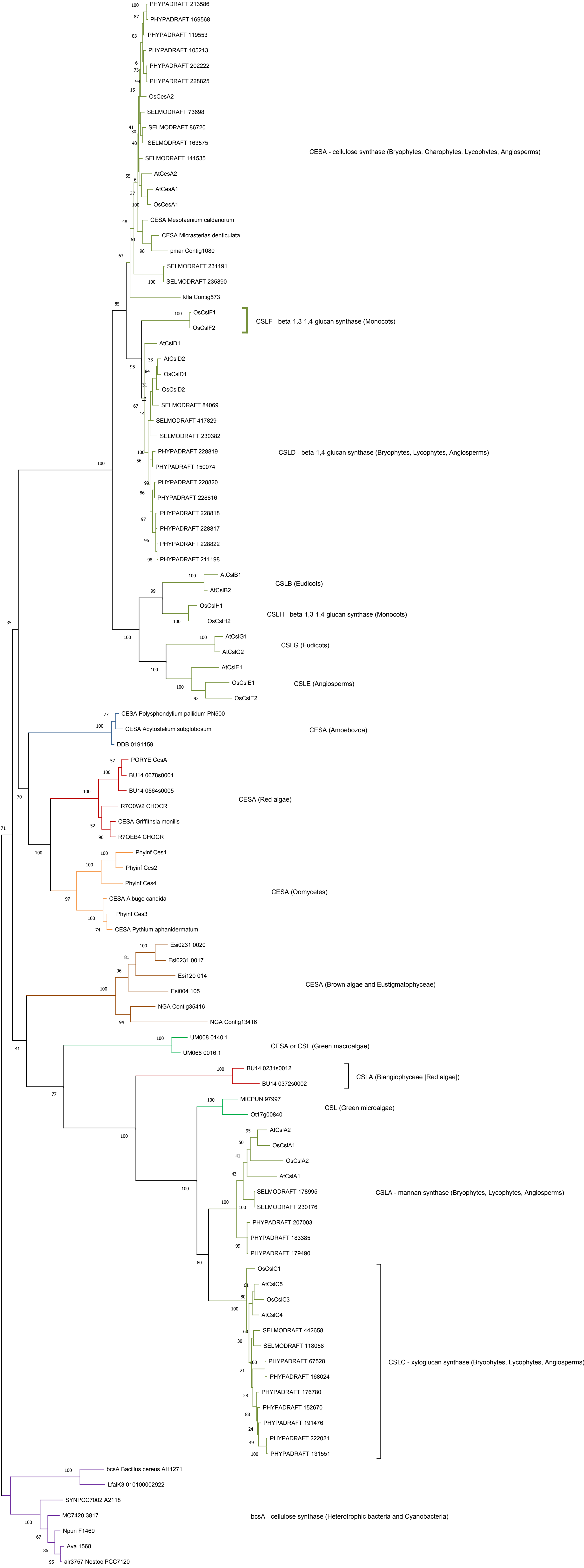

Supplement: Supplementary file 1 [file genes-12-01059-s001.zip › Supplementary files/Supplementary_Figure S1.pdf]

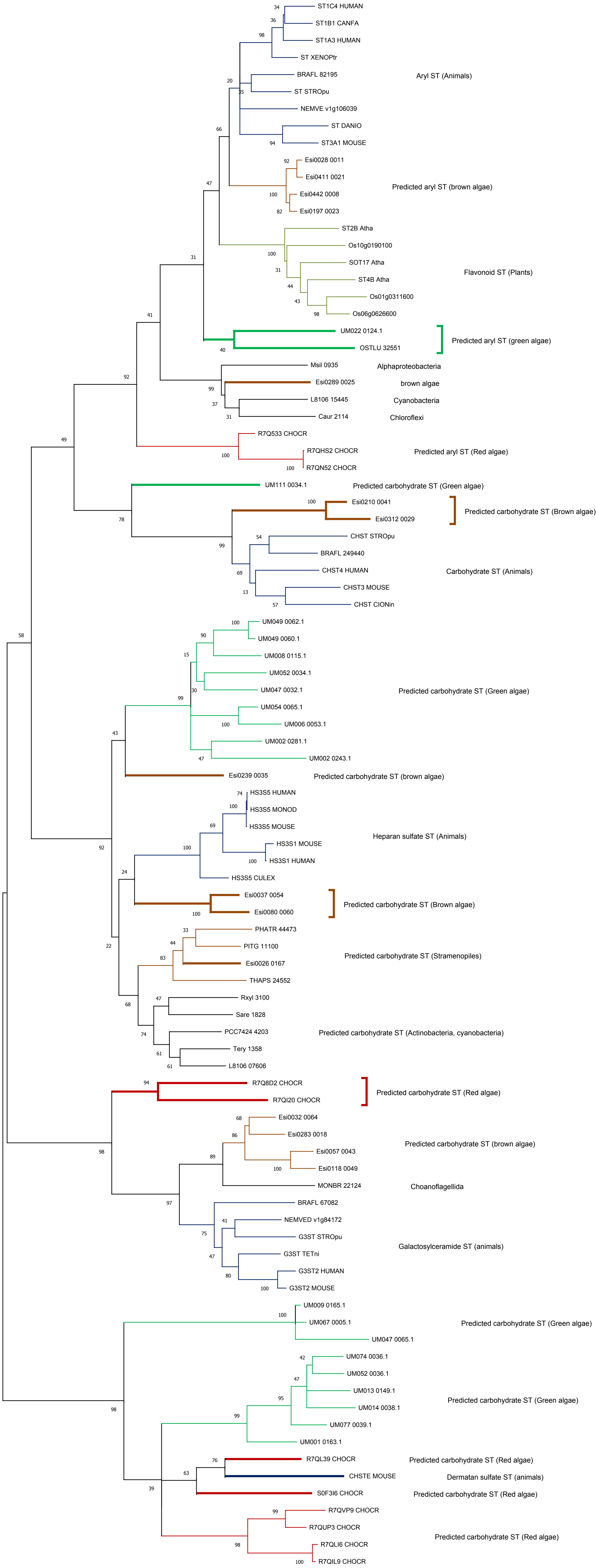

Supplement: Supplementary file 1 [file genes-12-01059-s001.zip › Supplementary files/Supplementary_Figure S2.pdf]
